# Longitudinal Study of Transcriptomic Changes Occurring over Six Weeks of CHOP Treatment in Canine Lymphoma Identifies Prognostic Subtypes

**DOI:** 10.3390/vetsci11110540

**Published:** 2024-11-05

**Authors:** Miles W. Mee, Sydney Faulkner, Geoffrey A. Wood, J. Paul Woods, Dorothee Bienzle, Brenda L. Coomber

**Affiliations:** 1 Department of Biomedical Sciences, Ontario Veterinary College, University of Guelph, Guelph, ON N1G 2W1, Canada; 2 Department of Pathobiology, Ontario Veterinary College, University of Guelph, Guelph, ON N1G 2W1, Canada; 3 Department of Clinical Studies and Mona Campbell Center for Animal Cancer, Ontario Veterinary College, University of Guelph, Guelph, ON N1G 2W1, Canada

**Keywords:** dog, RNA-Seq, hemolymphatic disorders, gene expression, cancer

## Abstract

The majority of canine lymphoma patients treated with the standard of care, the CHOP chemotherapy protocol, initially achieve remission but eventually relapse with a multi-drug-resistant phenotype. This study assesses gene expression profiles of canine lymphoma tumor cell populations using RNA-Seq data from 15 matched patient samples taken prior to treatment and again six weeks into treatment with CHOP. Two distinct clusters were present in the t-SNE dimensionality reduction of the gene expression profiles. There was a significant difference in progression-free survival (PFS) between the cluster groups, with a median of 43.5 days in a group of six patients and 185 days in another group of nine patients. Comparing the group with shorter PFS to the group with longer PFS, we identified 265 significantly enriched GO:BP terms in 3874 significantly up-regulated genes and 740 significantly enriched GO:BP terms in 3236 significantly down-regulated genes. Comparing the six-week timepoint against the initial timepoint, in the group with longer PFS, we identified 277 significantly enriched GO:BP terms in 413 significantly up-regulated genes and 222 significantly enriched GO:BP terms in 267 significantly down-regulated genes. In the group with shorter PFS, we only identified 27 significantly differentially expressed genes, for this comparison. We found DNA damage response genes to be enriched in the down-regulated genes in both comparisons. These results identify and characterize two transcriptionally distinct groups of canine lymphoma patients with significantly different responses to CHOP chemotherapy.

## Simple Summary

The standard of care for canine lymphoma is the CHOP chemotherapy protocol. The majority of patients initially achieve remission but eventually relapse with a multi-drug-resistant phenotype. We identified two transcriptionally distinct groups of canine lymphoma with a significant difference in progression-free survival. We describe the transcriptional differences between the two groups and how their transcription profiles change over the course of six weeks of CHOP treatment. These results contribute to the understanding of how canine lymphoma tumor cell populations respond to CHOP therapy.

## 1. Introduction

Canine lymphoma (cL) is the most common hemolymphatic neoplasm in dogs, occurring at an estimated incidence of about 20–100 per 100,000 dogs [1,2,3]. Both canine and human non-Hodgkin lymphoma have similar clinical presentation, molecular biology, therapy, and treatment response, making dogs an effective comparative model [4,5,6]. The World Health Organization classification of human lymphoma uses morphology, topography, immunophenotype, and clinical progression to define more than 30 distinct subtypes [7]. This classification scheme has been modified for canine lymphomas [8]. More broadly, three main prognostic molecular subgroups of canine lymphoma have been identified [9]. Ordered from worst to best prognosis, they include high-grade T-cell, B-cell lymphoma, and low-grade T-cell lymphoma [9].

The treatment for both human and canine lymphoma is mostly standard across subtypes, consisting of a multi-agent chemotherapy protocol of Cyclophosphamide, Hydroxydaunorubicin (doxorubicin), vincristine (Oncovin), and Prednisone (CHOP), with the addition of Rituximab in humans [10,11]. Generally, CHOP causes cell arrest and apoptosis by damaging DNA [12,13,14,15]. Variations in timing and dosage of CHOP have been explored [16]. Other chemotherapies and combinations of chemotherapies, including with agents of CHOP, have also been explored and reviewed [17]; however, CHOP remains the most common treatment for cL. Most cL patients have a high initial response rate to CHOP, with 70% to 85% of dogs achieving remission [18,19]. The duration of remission for cL patients treated with CHOP varies, with a median period of seven to ten months, but the majority of patients relapse and die within two years [18,19].

Many cancers, including cL, respond to chemotherapy treatment initially but relapse with a multi-drug-resistant phenotype [20]. When introduced to treatment-naive, heterogeneous tumor cell populations, the selective pressure of chemotherapeutic agents results in clonal evolution [21]. Chemoresistant subclones within the larger chemosensitive tumor cell population continue to proliferate despite exposure to chemotherapeutic agents, overtaking the initial tumor cell population and eventually resulting in multi-drug-resistant relapse [21]. Identifying mutations and gene expression profiles that are drivers of multi-drug resistance and relapse are active areas of research [20,21,22,23].

Various RNA sequencing (RNA-Seq) and microarray studies of cL gene expression have focused on topics including molecular subtyping [9], tumor-normal comparison [24], chemoresistant–chemosensitive comparison [25], identification of single nucleotide variants [26], and copy number variations [27]. Gene expression changes occurring in cL cell populations in response to CHOP treatment have not yet been fully investigated. Characterizing these gene expression differences is important for understanding treatment-resistant relapse in cL. This study compares gene transcript abundances of matched samples from 15 cL patients taken prior to treatment and six weeks into CHOP treatment.

## 2. Materials and Methods

### 2.1. Case Enrolment

Dogs diagnosed by cytology or histology with lymphoma, who had received no prior treatment other than a single injection of prednisone, were eligible for this study. There were no breed, sex, or age restrictions, but dogs with other concurrent neoplasms or prior neoplasms including lymphoma were excluded. The lymphomas were immunophenotyped by flow cytometry as described previously [28]. This study was a prospective analysis of consecutively recruited cases over the period of recruitment. Of all initially recruited cases, some were not suitable for further analysis due to insufficient yield of high-quality RNA from the initial timepoint sample, the six-week timepoint sample, both samples, or patient withdrawal. Dogs were enrolled with the intention to be treated with standard CHOP therapy and were monitored for a minimum of six months to assess progression-free survival (PFS). Informed consent for this study was obtained from all clients prior to enrollment and sample collection. This study was conducted in accordance with the Canadian Council on Animal Care (CCAC) Guidelines, as approved and supervised by the University of Guelph Animal Care Committee.

### 2.2. RNA-Seq Data Generation and Processing

Tumor samples were collected immediately prior to treatment and six weeks into treatment from the enlarged lymph nodes of cL patients by fine needle aspiration using multiple passes of sterile 22-gauge needles connected to a 6 mL syringe. Parallel samples were also collected for cytosmears and for immunophenotyping. The samples for gene expression analysis were directly expressed into sterile collection vials containing 1.0 mL of RNAprotect Cell Reagent (Qiagen; Toronto, ON, Canada). Samples were stored at room temperature overnight and then at −80 °C prior to RNA isolation. Poly A-RNA was isolated from the tissue samples using the QIAGEN RNeasy isolation kit (Toronto, ON, Canada) as described in the RNeasy instruction book without modification and quantified using a Thermo Scientific Nanodrop spectrophotometer (Toronto, ON, Canada). The RNA integrity number (RIN) was calculated for each sample using an Agilent 2100 Bioanalyzer (Santa Clara, CA, USA). Samples with an RIN above 9 were selected for sequencing. The selected samples were sequenced using the Illumina NextSeq (San Diego, CA, USA) platform by the London Regional Genomics Centre, London, ON, Canada. The sequence data were returned in single read, 75 bp, FASTQ format. The RNA sequencing results for this publication have been deposited in NCBI’s Gene Expression Omnibus and are accessible through GEO Series accession number GSE179920 (https://www.ncbi.nlm.nih.gov/geo/query/acc.cgi?acc=GSE179920, accessed on 29 October 2024). The quality of RNA-Seq reads was assessed using FastQC [29]. The reference genome (CanFam3.1) and corresponding gene annotation file (CanFam3.1.104.gtf) were downloaded from Ensembl [30]. The raw FASTQ reads were aligned to the CanFam3.1 reference genome using Hisat2 [31], generating Binary Alignment Map (BAM) files. The BAM files were then used as input for transcript assembly with StringTie [32], producing transcript annotations in Gene Transfer Format (GTF) files. Raw read counts were extracted from the assembled GTF files using the prepDE.py script associated with the Ballgown R package [33], which consolidated transcript counts across multiple samples into a single table of raw transcript counts from all the samples. Dimensionality reduction of transcript counts was performed using the Rtsne package in R.

### 2.3. Survival Analysis

Kaplan-Meier log-rank analysis of patient progression-free survival clinical data was performed using the Survival and Survminer R packages.

### 2.4. Differential Gene Expression

RNA-Seq raw transcript counts for 30,158 canine Ensembl IDs, from samples at both timepoints, were used as input. The genes were filtered for genes with ‘gene_biotype’ of ‘protein_coding’ in the CanFam3.1.104.gtf annotation file, leaving 10,968 genes. Gene differential expression was evaluated using the DESeq2 R package [34]. For the six-week vs. initial timepoint comparison, a factor representing the donor from which each sample was obtained was included in the DESeq2 design formula, allowing the model to account for individual variability between donors. The Benjamini–Hochberg method was used to adjust the resulting DESeq2 *p*-values for false discovery rate (FDR) [35]. For further analysis, the results were filtered for genes with a ‘baseMean’ greater than 50.

### 2.5. Enrichment Analysis

The gene symbols of the significantly up-regulated and down-regulated genes (DESeq2 FDR-adjusted *p*-value < 0.05) were separately used as input for enrichment analysis. g:Profiler was used to test differentially expressed gene lists for the enrichment of Gene Ontology (GO) terms, including biological process (GO:BP), cellular compartment (GO:CC), and molecular function (GO:MF), and for the enrichment of TRANSFAC transcription factor motifs [36]. The human GO Gene Matrix Transposed (GMT) file for GO term gene lists was used to test enrichment, as annotations in humans are more well characterized compared to annotations for animals. The significantly enriched terms (FDR-adjusted *p*-value < 0.05) were visualized using Cytoscape and the Enrichment Map plugin [37].

## 3. Results

### 3.1. Clustering and Survival

**Figure 1:**
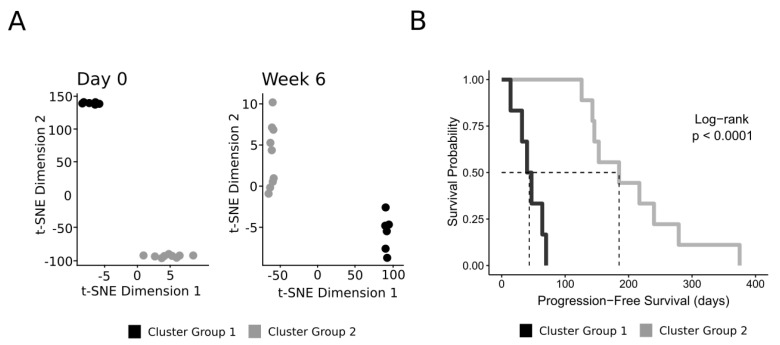
(**A**) t-SNE of transcript count gene expression data from samples from 15 dogs with lymphoma taken prior to treatment and six weeks into treatment with CHOP. The cluster of six patients is shown in black, and the cluster of nine patients is shown in gray. (**B**) Kaplan–Meier plot of PFS for the clusters shown in (**A**).

We analyzed bulk RNA-seq data from 15 cL patients taken at an initial pre-treatment timepoint and a timepoint six weeks into treatment with CHOP. The cohort clinicopathologic data are described in Table 1. The cohort included seven patients immunophenotyped as B-cell lymphoma and three patients immunophenotyped as T-cell lymphoma. Due to technical error, five patients did not undergo immunophenotyping and are included as ‘unknown immunophenotype’. We visualized the full gene expression profiles using t-SNE dimensionality reduction. There were two distinct clusters at both the initial and six-week timepoints, one cluster of six patients (Cluster Group 1) and another cluster of nine patients (Cluster Group 2) (Figure 1A). We compared PFS between the two groups using Kaplan-Meier log-rank analysis. There was a significant difference in PFS (*p*-value < 0.0001) between the two groups (Figure 1B). The cluster group with six patients had a median PFS of 43.5 days, and the group with nine patients had a median PFS of 185 days (Figure 1B). Patient characteristics are described in Table 2. All patients from the cluster group with shorter PFS had undergone progression before any of the patients from the cluster group with longer PFS progressed (Figure 1B). There were three B-cell, one T-cell, and two unknown immunophenotype patients in the shorter PFS cluster group, and there were four B-cell, two T-cell, and three unknown immunophenotype patients in the cluster group with longer PFS (Table 2). In the cluster group with shorter PFS, there were three patients with stage III and three patients with stage V lymphoma, and in the cluster group with longer PFS, there were five patients with stage III, two patients with stage IV, and two patients with stage V lymphoma (Table 2).

### 3.2. Cluster Group 1 vs. Cluster Group 2 at Initial and Six-Week Timepoints

**Figure 2:**
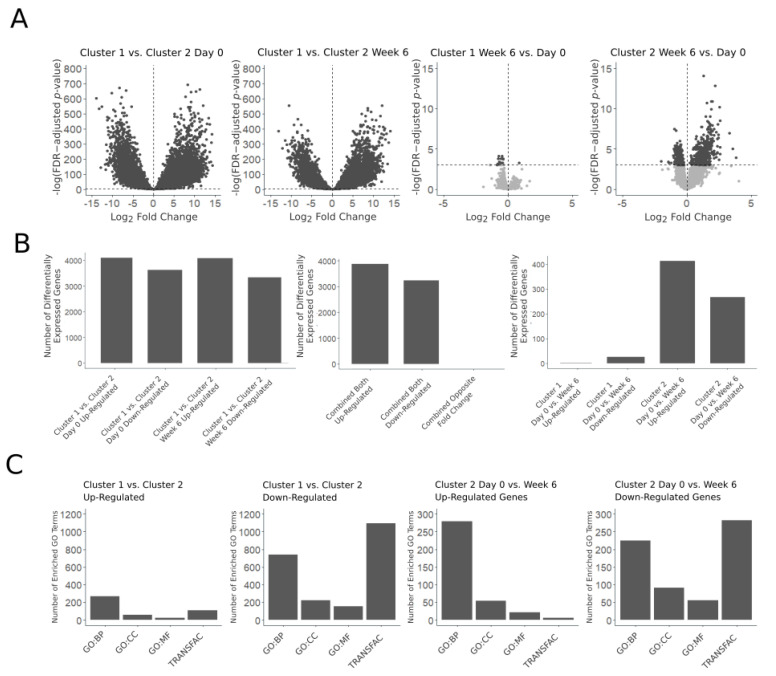
(**A**) Volcano plots showing DESeq2 differential gene expression results comparing the two cluster groups at the initial timepoint, comparing the two cluster groups at the six-week timepoint, comparing Cluster Group 1 at the six-week timepoint to the initial timepoint, and comparing Cluster Group 2 at the six-week timepoint to the initial timepoint. Significantly differentially expressed genes are shown in dark gray, and non-significantly differentially expressed genes are shown in light gray. (**B**) Bar charts showing the number of differentially expressed genes from the comparisons in (**A**). (**C**) Bar charts showing the number of significantly enriched terms in the differentially expressed genes in (**A**).

**Figure 3:**
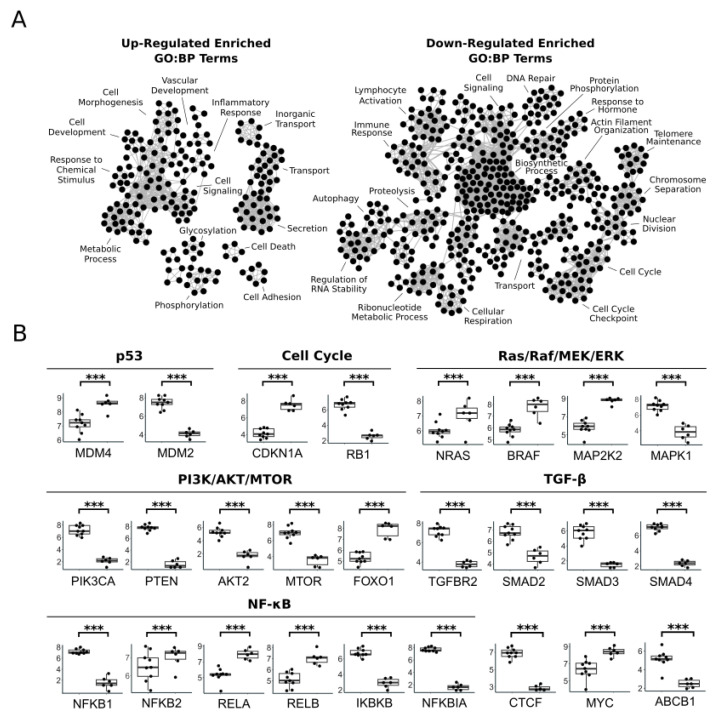
(**A**) Enrichment maps showing select significantly enriched GO:BP terms in the list of up-regulated and down-regulated significantly differentially expressed genes comparing the two clusters at the initial timepoint and at the six-week timepoint. Nodes in the network represent significantly enriched GO:BP terms, and edges represent shared genes between the gene sets of the terms the edge connects. (**B**) Boxplots showing log normalized transcript counts from the initial timepoint for specific differentially expressed genes. Cluster Group 1 is shown on the right, and Cluster Group 2 is shown on the left in each box plot. DESeq2 FDR-adjusted *p*-value < 0.0001 (***).

We compared the gene expression profiles between the two cluster groups at the initial timepoint and at the six-week timepoint. At the initial timepoint, we found 7721 significantly differentially expressed genes (FDR-adjusted *p*-value < 0.05), 4095 up-regulated, and 3626 down-regulated (Figure 2A,B; Table S1). At the six-week timepoint, we found 7416 significantly differentially expressed genes (FDR-adjusted *p*-value < 0.05), 4077 up-regulated, and 3339 down-regulated (Figure 2A,B; Table S2). Of the significantly differentially expressed genes, 3874 up-regulated genes and 3236 down-regulated genes were significant at both timepoints, which accounts for about 85% of the genes tested (Figure 2B). No genes were significantly differentially expressed at both timepoints with opposite fold changes. We identified significantly enriched GO terms (FDR-adjusted *p*-value < 0.05) separately in the up- and down-regulated, significantly differentially expressed genes at both timepoints. In the genes up-regulated at both timepoints, we identified 265 GO:BP, 59 GO:CC, and 23 GO:MF significantly enriched terms, and 112 significantly enriched TRANSFAC transcription factor motif terms (Figure 2C; Table S3). The enriched GO:BP terms included terms related to signaling, response to chemical stimulus, secretion, transport, metabolic process, vascular development, cell adhesion, and phosphorylation (Figure 3A). In the genes down-regulated at both timepoints, we identified 740 GO:BP, 223 GO:CC, and 156 GO:MF significantly enriched terms, and 1091 significantly enriched TRANSFAC transcription factor motif terms (Figure 2C; Table S4). The enriched GO:BP terms in the down-regulated genes included terms related to lymphocyte activation, immune response, proteolysis, autophagy, regulation of RNA stability, DNA repair, chromosome separation, and cell cycle checkpoint (Figure 3A).

Several important genes and pathways were significantly differentially expressed (FDR-adjusted *p*-value < 0.05) in the cluster group with shorter PFS compared to the cluster group with longer PFS (Figure 3B). The p53 regulators MDM4 and MDM2 were up-regulated and down-regulated, respectively. Cell cycle regulators CDKN1A and RB1 were up-regulated and down-regulated, respectively. Ras/Raf/MEK/ERK pathway genes NRAS, BRAF, ARAF, and MAPK2 were up-regulated but MAPK1 was down-regulated. PI3K/AKT/MTOR pathway genes PIK3CA, PTEN, AKT2, and MTOR were down-regulated. AKT target genes TSC1, BAD, FOXO1, and CDKN1A were up-regulated and HIF1A, GSK3B, FOXO1, and MDM2 were down-regulated. TGF-β pathway genes TGFBR2, SMAD2, SMAD3, and SMAD4 were down-regulated. NF-κB pathway genes NFKB2, RELA, and RELB were up-regulated and NFKB1, IKBKB, and NFKBIA were down-regulated. MYC transcription factor was up-regulated. The chromatin organization gene CTCF was down-regulated. The multi-drug resistance gene ABCB1 was down-regulated. Since only about 10% of the genes tested, 829 genes, were not significantly differentially expressed between the two cluster groups at both timepoints, these genes may be of interest as well in that their level of expression may be necessary for cellular function of both molecularly distinct subtypes. Biologically important genes with no significant difference in expression between the two groups included TP53, CDK6, AKT1, APC, LEF1, and TERT.

### 3.3. Cluster Group 1 Six-Week vs. Initial Timepoint and Cluster Group 2 Six-Week vs. Initial Timepoint

**Figure 4:**
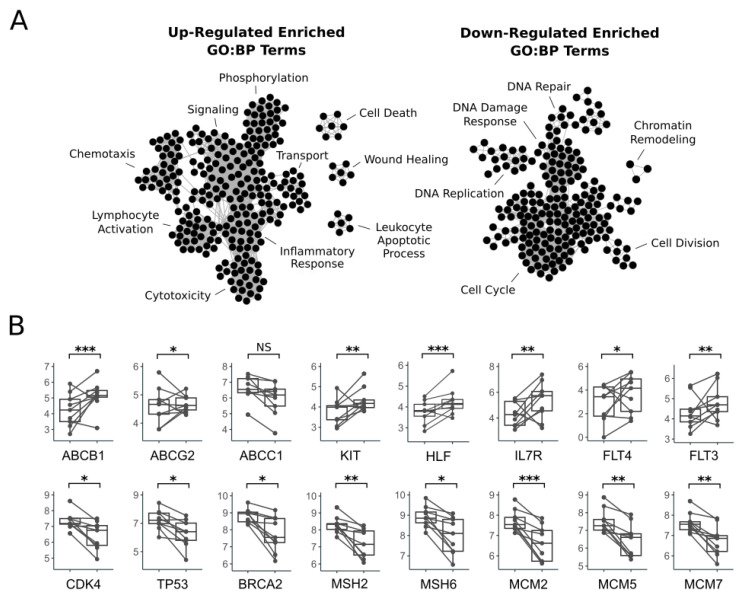
(**A**) Enrichment maps showing select significantly enriched GO:BP terms in the lists of up-regulated and down-regulated significantly differentially expressed genes comparing Cluster Group 2 at the six-week timepoint to the initial timepoint. Nodes in the network represent significantly enriched GO:BP terms, and edges represent shared genes between the gene sets of the terms the edge connects. (**B**) Boxplots showing log normalized transcript counts for specific differentially expressed genes. The initial timepoint is shown on the left, and the six-week timepoint is shown on the right in each box plot. The expression values for each patient are connected between the timepoints. DESeq2 FDR-adjusted *p*-value < 0.05 (*), < 0.001 (**), < 0.0001 (***), > 0.05 non-significant (NS).

In the cluster group with shorter PFS, comparing the six-week timepoint to the initial timepoint, there were 27 significantly differentially expressed genes (FDR-adjusted *p*-value < 0.05), 2 up-regulated, and 25 down-regulated (Figure 2A,B; Table S5). In the cluster group with longer PFS, comparing the six-week timepoint to the initial timepoint, there were 680 significantly differentially expressed genes (FDR-adjusted *p*-value < 0.05), 413 up-regulated, and 267 down-regulated (Figure 2A,B; Table S6). We identified significantly enriched GO terms (FDR-adjusted *p*-value < 0.05) separately in the up- and down-regulated significantly differentially expressed genes. In the up-regulated genes, we identified 277 GO:BP, 58 GO:CC, and 25 GO:MF significantly enriched terms, and six significantly enriched TRANSFAC transcription factor motif terms (Figure 2C; Table S7). The enriched GO:BP terms in the up-regulated genes included terms related to signaling, chemotaxis, lymphocyte activation, cytotoxicity, inflammatory response, and transport (Figure 4A). Notably, the ATP-binding cassette transporter multi-drug resistance-related genes ABCB1 and ABCG2 were significantly up-regulated (Figure 4B). Among the significantly down-regulated genes, we identified 222 GO:BP, 92 GO:CC, and 55 GO:MF significantly enriched terms, and 275 significantly enriched TRANSFAC transcription factor motif terms (Figure 2C; Table S8). The enriched GO:BP terms in the down-regulated genes included terms related to DNA damage response, and cell cycle (Figure 4A). Down-regulated DNA damage response genes included TP53, BRCA2, MSH2, MSH6, MCM2, MCM5, and MCM7 (Figure 3B).

## 4. Discussion

The failure of many cL patients treated with CHOP therapy to achieve long-term remission has raised interest in understanding how cL acquires resistance over the course of treatment and what differentiates patients who do not initially respond to treatment from those that do. In this study, we identify two distinct clusters of transcriptional profiles in which the patients have significantly different PFS between the two clusters. We describe the significant gene expression differences between the cluster groups as well as the changes in the transcriptional profiles occurring in the tumor cell populations of these cluster groups over the course of a six-week round of treatment with CHOP. Although we have a small sample size of 15 patients with only three T-cell samples, there were T-cell, B-cell, and unknown immunophenotype samples in both cluster groups, providing some evidence that the observed clusters are independent of cell type of origin. We also found stage III and stage V lymphomas in both clusters, suggesting that the cluster group with shorter PFS was not associated with higher-stage lymphomas. The cluster group with shorter PFS had few differentially expressed genes at the six-week timepoint compared to the initial timepoint, suggesting there was not a significant amount of tumor cell population evolution driven by selective pressure of CHOP. In contrast, the differentially expressed genes and enriched pathways in the comparison of the six-week to initial timepoint for the cluster group with longer PFS describe the effect of the selective pressure of CHOP treatment on the tumor cell population.

The distinct clustering of gene expression profiles, significant difference in PFS between clusters, large percent of the genome differentially expressed between the cluster groups at both timepoints, enrichment of relevant pathways in the differentially expressed genes, and differential expression of biologically important genes with a high level of significance and large fold changes suggest the lymphomas in the two cluster groups are very different on a molecular level. Several key molecular pathways were differentially regulated between the cluster groups. These differentially regulated pathways provide potential mechanisms for the shorter PFS cluster group resisting treatment with CHOP.

### 4.1. p53 Regulation

MDM4 and MDM2 are key regulators of p53, one of the most important proteins in arresting the cell cycle in response to DNA damage [38]. MDM4 inhibits p53 activity and MDM2 degrades p53, but upon DNA damage, MDM2 can degrade itself and MDM4, resulting in p53 stabilization and activation, promoting cell cycle arrest and apoptosis [39]. Although TP53 itself was not significantly differentially expressed, the up-regulation of MDM4 and down-regulation of MDM2 in the cluster group with shorter PFS could be resulting in inhibition of p53, allowing evasion of DNA damage-related apoptosis.

### 4.2. Cell Cycle Regulation

Cyclin-dependent kinases (CDKs) are the kinases that regulate cell cycle progression, and cyclins are the regulatory subunits that bind to CDKs, activating them [40,41]. In the cluster group with shorter PFS, CCND1 (Cyclin D1), CCNA1 (Cyclin A1), CCNB2 (Cyclin B2), CDK4, and CDK2 were up-regulated and CCND2 (Cyclin D2), CCNE2 (Cyclin E2), CCNA2 (Cyclin A2), and CDK1 were down-regulated, suggesting a complex selective regulation of the cell cycle. CDKN1A (p21) and RB1 (pRB) are key inhibitory regulators of the cell cycle and response to DNA damage [40,41]. CDKN1A was up-regulated and RB1 was down-regulated in the cluster group with shorter PFS. CDKN1A is a CDK inhibitor that halts the cycle in the G1 phase in response to DNA damage [42]. RB1 inhibits the cell cycle progression from the G1 phase to the S phase by binding and sequestering E2F transcription factors. Down-regulation of RB1 in the cluster group with shorter PFS could less effectively sequester E2F transcription factors, resulting in transcription of genes necessary for S phase entry, thereby promoting cell cycle progression despite potential DNA damage [43].

### 4.3. PI3K/AKT/MTOR Pathway

The PI3K/AKT/mTOR signaling pathway is a key regulator of cell growth, metabolism, survival, proliferation, and angiogenesis [44,45]. In the cluster group with shorter PFS, members of the PI3K/AKT/MTOR pathway, including PIK3CA, PTEN, AKT2, and MTOR, were down-regulated. PIK3CA encodes the catalytic subunit of phosphoinositide 3-kinase (PI3K), which phosphorylates phosphatidylinositol-3,4,5-trisphosphate (PIP3), which in turn recruits and activates AKT2 [44,45]. AKT2 has a wide range of targets, which include activation of HIF1A, IKK, and MDM2 and inhibition of the TSC1/TSC2 complex, BAD, GSK3B, FOXO1, and CDKN1A [46]. TSC1, BAD, FOXO1, and CDKN1A were up-regulated, and HIF1A, GSK3B, MDM2, and FOXO1 were down-regulated in the cluster group with shorter PFS. AKT2 activates MTOR through its inhibition of the TSC1/TSC2 complex, which inhibits MTOR. MTOR is a central kinase that regulates protein synthesis, cell growth, and metabolism [47]. PTEN acts as a negative regulator of the PI3K/AKT pathway, but its down-regulation here may be due to the broader suppression of the whole pathway [44,45]. PI3K/AKT/mTOR is often up-regulated in aggressive cancers but is down-regulated in the cluster group with shorter PFS.

### 4.4. Ras/Raf/MEK/ERK Pathway

The Ras/Raf/MEK/ERK signaling cascade is a key pathway involved in cell proliferation, differentiation, and survival [48,49]. Some members of the Ras/Raf/MEK/ERK signaling pathway were up-regulated in the cluster group with shorter PFS, including Ras gene NRAS, Raf genes BRAF and ARAF, and MEK gene MAP2K2, while others, including Ras gene KRAS, Raf gene RAF1, and ERK gene MAPK1, were down-regulated. The up-regulation and down-regulation of members of the Ras/Raf/MEK/ERK signaling pathway reflects a complex dysregulation of the pathway, but the up-regulation of Ras, Raf, and MEK genes could lead to increased phosphorylation and activation of MAPK1 even if the protein levels are lower.

### 4.5. TGF-β Pathway

The TGF-β signaling pathway regulates cellular processes such as proliferation, apoptosis, and differentiation [50,51,52]. TGF-β acts as a tumor suppressor by preventing uncontrolled cell growth [50,51,52]. In the cluster group with shorter PFS, members of the TGF-β signaling pathway were down-regulated, including TGF-β receptor TGFBR2, as well as SMAD2, SMAD3, and SMAD4. TGFBR2 recruits and phosphorylates TGFBR1 [50,51,52]. This receptor complex subsequently phosphorylates SMAD2 and SMAD3, which form complexes with SMAD4 [50,51,52]. Upon translocation to the nucleus, these complexes regulate the transcription of target genes, which inhibit cell proliferation and induce apoptosis [50,51,52]. The down-regulation of TGF-β in the cluster group with worse PFS could lead to uncontrolled cellular proliferation and evasion of apoptosis.

### 4.6. NF-κB Pathway

The NF-κB pathway is a key regulator of immune response and inflammation [53,54,55,56]. In the cluster group with shorter PFS, members of the NF-κB pathway RELA (p65), RELB, and NFKB2 (p52) were up-regulated and NFKB1 (p50) was down-regulated. In the canonical NF-κB signaling pathway, dimers of p65 and p50 translocate to the nucleus to drive gene expression. This same activity also occurs with dimers of RelB and p52 in the alternative NF-κB pathway [53,54,55,56]. The activation of the canonical pathway promotes immune response, and activation of the alternative pathway promotes inflammation [53,54,55,56]. NFKBIA (IκBα) and IKBKB (IKKβ) were both down-regulated in the cluster group with shorter PFS. IκBα sequesters NF-κB in the cytoplasm, preventing its activation [53,54,55,56]. IKKβ phosphorylates IκBα, targeting it for degradation, allowing NF-κB dimers to translocate to the nucleus [53,54,55,56]. The up-regulation of RELB, NFKB2, and down-regulation of NFKBIA suggest activation of the alternative NF-κB pathway. The down-regulation of NFKB1 suggests the canonical pathway is down-regulated. The combination of up-regulated alternative NF-κB signaling and down-regulated canonical signaling could lead to a dynamic where the immune response is dysregulated and inflammation is promoted. In support of this dynamic, in the cluster group with shorter PFS, inflammatory response-related GO:BP terms were enriched in the up-regulated genes and immune response-related GO:BP terms were enriched in the down-regulated genes.

### 4.7. Chromatin Organization

CTCF plays a critical role in organizing the 3D structure of chromatin by binding to specific DNA sequences, creating boundaries, and forming loops in the chromatin structure, separating different chromatin domains [57,58]. This organization creates topologically associated domains, bringing distant regulatory elements into close proximity or separating them to prevent inappropriate interaction [57,58]. CTCF is essential for the proper regulation of gene expression, and the down-regulation of CTCF in the cluster group with shorter PFS is likely resulting in widespread disorganization of the chromatin structure and aberrant gene expression in the lymphomas of these patients.

### 4.8. DNA Damage Response

For both the comparisons (the cluster group with shorter PFS compared to the cluster group with longer PFS and the cluster group with longer PFS at the six-week timepoint compared to the initial timepoint), GO:BP terms related to DNA damage response were enriched in the down-regulated genes. DNA damage normally results in either cell cycle arrest to allow DNA repair mechanisms to repair the DNA damage before continuing through the cell cycle or apoptosis if the damage cannot be repaired. Many chemotherapeutic drugs, including agents of CHOP, induce apoptosis by damaging DNA. Each agent in CHOP has a unique mechanism of action. Cyclophosphamide forms cross-links both between and within DNA strands [12]. Doxorubicin stabilizes the topoisomerase II complex during replication, preventing the DNA double helix from being resealed [13]. Vincristine binds to the tubulin protein, preventing the formation of microtubules and interfering with chromosome separation during metaphase [14]. Prednisone is a glucocorticoid that induces apoptosis [15]. Deficiencies in DNA damage response can contribute to chemoresistance if the cancer cell cycle fails to be arrested in response to the DNA damage inflicted by the chemotherapeutics, thereby allowing the cells to proceed through the cell cycle unrepaired [59]. The genes related to DNA damage were driving the enrichment of the TRANSFAC transcription factor motif terms in both comparisons, suggesting that the gene expression changes in these genes could be linked through transcription factors. In the cluster group with longer PFS, the enrichment of GO:BP terms related to DNA damage response in the genes down-regulated at the six-week timepoint compared to the initial timepoint suggests the selective pressure of CHOP treatment on the tumor cell population selects for cells with reduced DNA damage response and further suggests down-regulated DNA damage response is a driver of resistance to CHOP in the cluster group with shorter PFS.

### 4.9. ATP Binding Cassette (ABC) Transporters

The up-regulation of members of the ATP binding cassette (ABC) transporter family is an important contributor to multi-drug resistance, as these molecules are able to efflux chemotherapeutic compounds from cancer cells [60,61]. The most important ABC transporters related to drug resistance include ABCB1 (MDR1/P-glycoprotein), ABCC1 (MRP1), and ABCG2 (BCRP) [60,61]. ABCB1 was significantly up-regulated in the cluster group with longer PFS at the six-week timepoint compared to the initial pre-treatment timepoint. ABCB1 is a known cause of chemoresistance and relapse in cL [62]. Increasing ABCB1 expression has been shown to induce resistance to CHOP members doxorubicin and vincristine in cL cell lines [63]. A longitudinal study associated multi-drug resistance with increased ABCB1 expression in B-cell cL and increased ABCG2 expression in T-cell cL [64]. Efforts to develop ABCB1 as a therapeutic target in cL have been mostly unsuccessful. PSC-833, an inhibitor of ABCB1, has shown to be effective in reducing resistance to doxorubicin and vincristine in cL cell lines but failed to prolong remission in a clinical setting [65,66]. L-asparaginase is not targeted by ABCB1 for efflux and is often added to cL rescue protocols; however, studies show variable effectiveness in clinical settings [67,68]. The up-regulation of ABCB1 in the tumor cell population of the cluster group with longer PFS at the six-week timepoint compared to the initial timepoint supports previous research implicating ABCB1 in acquired multi-drug resistance and relapse in cL. In contrast, the cluster group with shorter PFS had down-regulated ABCB1 compared to the cluster group with longer PFS at both timepoints, suggesting drug efflux via ABCB1 is not a mechanism of treatment resistance in the shorter PFS cluster group.

## 5. Conclusions

The results presented here highlight biological processes involved in CHOP treatment resistance in cL. Our findings identify and characterize two transcriptionally distinct groups of cL patients with significantly different responses to CHOP chemotherapy. The clear differentiation in gene expression profiles of the groups at the initial pre-treatment timepoint suggests these results could have clinical significance in identifying patients who will not respond well to CHOP therapy before treatment starts. The significantly differentially expressed genes described here could inform the development of clinical tests based on detection at the gene expression level with qPCR, microarrays, or detection at the protein expression level with immunohistochemistry, enzyme-linked immunosorbent assays, or Western blotting. The ability to detect these patients prior to initiation of chemotherapy could allow for the employment of alternative treatments, possibly targeting the pathways described in this study. However, it is not clear specifically which of the several pathways we have identified are most responsible for the differences in CHOP response. As well, a lack of current therapies to target many of the identified pathways is a major challenge in moving our findings into clinical practice. In the short term, it may be practical to identify a limited number of genes that can be employed in such rapid biomarker tests to stratify patients away from CHOP to first-line rescue chemotherapy. Furthermore, identifying patients who will respond poorly to CHOP could allow for more accurate prognosis determination, which may affect decisions about the costs/benefits of treatment.

This study is primarily limited by its small sample size and also by limited knowledge of sample subtypes. Future studies of larger patient cohorts and complete immunophenotyping could further validate the existence of these subtypes and determine whether the subtype with shorter PFS corresponds to one or more known subtypes of lymphoma. More detailed analysis of how the observed differences relate to lymphoma subtypes (B-cell vs. T-cell) would also be valuable, as would comparison with human non-Hodgkin lymphoma. Future research could focus on investigating gene expression changes between the initial diagnosis and subsequent cycles of CHOP chemotherapy as an aid in identifying the development of CHOP resistance in initially responsive patients.

## Figures and Tables

**Table 1:** Demographic characteristics of the cohort of 15 dogs.

Characteristic		Number (%)
Age (years)		7.5 ± 2.1 (Mean ± SD)
Sex	Male	2 (13.3%)
	Neutered male	8 (53.3%)
	Female	0 (0%)
	Spayed female	5 (33.3%)
Breed	Mixed breed	3 (20%)
	Golden retriever	5 (33.3%)
	Labrador retriever	1 (6.7%)
	Mastiff	1 (6.7%)
	Dalmatian	1 (6.7%)
	Cocker spaniel	1 (6.7%)
	Standard poodle	1 (6.7%)
	Maltese terrier	1 (6.7%)
	Wire haired fox terrier	1 (6.7%)
Stage	III	8 (53.3%)
	IV	2 (13.3%)
	V	5 (33.3%)
Immunophenotype	B-cell	7 (46.7%)
	T-cell	3 (20%)
	Unknown	5 (33.3%)

**Table 2:** Sample clinicopathologic characteristics.

Cluster Group	Immunophenotype	Stage	PFS (Days)
1	B-cell	III	47
1	B-cell	III	32
1	B-cell	V	14
1	T-cell	III	70
1	Unknown	V	64
1	Unknown	V	40
2	B-cell	III	146
2	B-cell	IV	217
2	B-cell	V	185
2	B-cell	V	240
2	T-cell	III	143
2	T-cell	III	375
2	Unknown	III	126
2	Unknown	III	279
2	Unknown	IV	153

## Data Availability

The RNA sequencing dataset supporting the conclusions of this article is available in the NCBI’s Gene Expression Omnibus and is accessible through GEO Series accession number GSE179920 (https://www.ncbi.nlm.nih.gov/geo/query/acc.cgi?acc=GSE179920, accessed on 29 October 2024). Other data that support the conclusions of this article are included within the article and its additional files.

## Supplementary Materials

The following supporting information can be downloaded at: https://www.mdpi.com/article/10.3390/vetsci11110540/s1, Table S1: Cluster 1 vs. Cluster 2 initial timepoint DESeq2 results; Table S2: Cluster 1 vs. Cluster 2—6-week timepoint DESeq2 results; Table S3: Cluster 1 vs. Cluster 2 g:Profiler enrichment results of genes up-regulated at both timepoints; Table S4: Cluster 1 vs. Cluster 2 g:Profiler enrichment results of genes down-regulated at both timepoints; Table S5: Cluster 1—6-week vs. initial timepoint DESeq2 results; Table S6: Cluster 2—6-week vs. initial timepoint DESeq2 results; Table S7: Cluster 2—6-week vs. initial timepoint g:Profiler enrichment results of up-regulated genes; Table S8: Cluster 2—6-week vs. initial timepoint g:Profiler enrichment results of down-regulated genes.
